# Time-optimized feeding is beneficial without enforced fasting

**DOI:** 10.1098/rsob.210183

**Published:** 2021-10-06

**Authors:** Kevin P. Kelly, Kate L. J. Ellacott, Heidi Chen, Owen P. McGuinness, Carl Hirschie Johnson

**Affiliations:** ^1^ Department of Biological Sciences, Vanderbilt University, Nashville, TN 37235, USA; ^2^ Institute of Biomedical and Clinical Science, University of Exeter Medical School, Exeter, UK; ^3^ Department of Biostatistics, Vanderbilt University Medical Center, Nashville, TN, USA; ^4^ Department of Molecular Physiology and Biophysics, Vanderbilt University School of Medicine, Nashville, TN, USA

**Keywords:** time-restricted feeding, circadian rhythms, lipid metabolism, fasting, energy expenditure, respiratory quotient

## Abstract

Time-restricted feeding (TRF) studies underscore that *when* food is consumed during the daily cycle is important for weight gain/loss because the circadian clock rhythmically modulates metabolism. However, the interpretation of previous TRF studies has been confounded by study designs that introduced an extended period of enforced fasting. We introduce a novel time-optimized feeding (TOF) regimen that disentangles the effects of phase-dependent feeding from the effects of enforced fasting in mice, as well as providing a laboratory feeding protocol that more closely reflects the eating patterns of humans who usually have 24 hour access to food. Moreover, we test whether a sudden switch from ad libitum food access to TRF evokes a corticosterone (stress) response. Our data indicate that the timing of high-fat feeding under TOF allows most of the benefit of TRF without obligatory fasting or evoking a stress response. This benefit occurs through stable temporal coupling of carbohydrate/lipid oxidation with feeding. These results highlight that timing the ingestion of calorically dense foods to optimized daily phases will enhance lipid oxidation and thereby limit fat accumulation.

## Graphical abstract

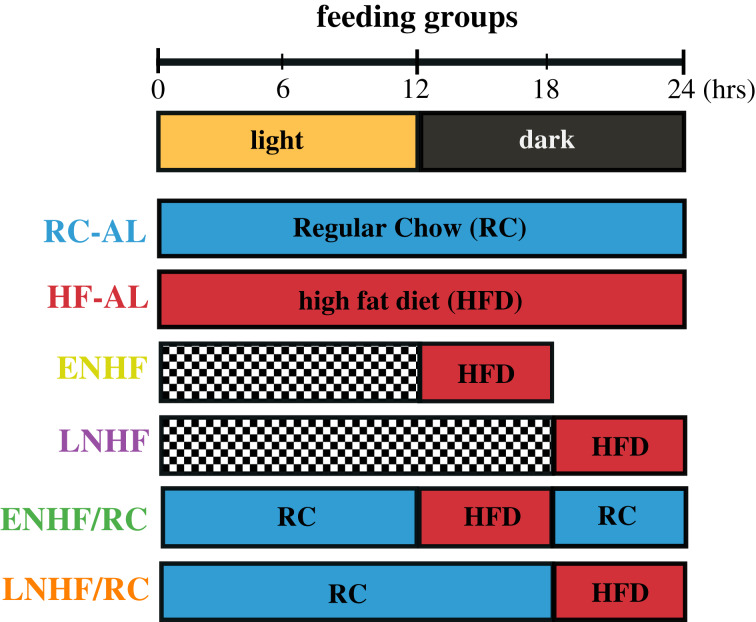

## 1. Introduction

The balance between weight gain and loss is largely determined by the quantity and quality of food consumed, as well as by the amount of physical activity. In addition, however, time-restricted feeding (TRF) studies underscore that another critical factor is *when* during the daily cycle that food is consumed. The circadian (daily) biological clock adapts organisms to the daily cycle, and it controls myriad biological processes, including metabolic pathways [1–4]. Consequently, metabolic rate and switching between preferred metabolic pathways is differentially regulated over the day/night cycle, which predicts that the daily timing of meals can affect nutrient disposition. A growing body of research in humans and model organisms confirms this prediction [1,3,5–7]. In particular, the daily timing of meals in humans can influence metabolic switching between lipid versus carbohydrate oxidation (CO) [8–11]. These findings and predictions have translational import, especially to shiftworkers whose daily timing of eating is disrupted and who are much more likely to develop obesity and metabolic syndrome-related disorders [3,5,6,12].

In model organisms such as mice, a commonly used experimental paradigm to illustrate the importance of daily meal timing is the TRF regimen [1,13–16]. In TRF, feeding is only allowed during defined intervals—frequently with preferred high-fat diet (HFD) chow—that are temporally alternated with intervals of enforced fasting in which no food is provided. One of the first studies to show that TRF regimens influence weight gain was a study with mice by Arble *et al.* [13], who restricted HFD feeding to a 12 h window in either the day (inactive) phase or in the night (active) phase of a LD 12 : 12 cycle (and 12 h fasting in the other interval of the LD 12 : 12 cycle). Arble *et al.* [13] reported that mice given HFD in their inactive (day) phase gained significantly more weight than mice given food in their active (night) phase, even though mice in both groups exhibited equivalent caloric intake and physical activity. Subsequent research by Hatori *et al.* [1] using a shorter allowed HFD feeding duration (8 h) in the night phase reported less weight gain as compared with HFD ad libitum (HF-AL) despite equivalent caloric intake and physical activity. Hatori *et al.* [1] concluded that the reduced weight gain of TRF was primarily due to the prolonged and absolute fasting period as a consequence of a shorter feeding period. Thus, they proposed that the reduced weight gain was due to a long daily fast (coupled with a shorter feeding duration). The attenuation of weight gain despite comparable food intake suggested that TRF increased energy expenditure (EE). Other TRF mouse studies have confirmed that time-restricted access to HFD coupled with enforced fasting has metabolic benefits, including improved insulin sensitivity, glucose tolerance, and liver triglyceride accumulation [17,18].

While the benefits of TRF seems to be clear in mice, studies of daily timing of meals in humans have reported conflicting results and interpretations. Some studies observed changes in EE based on daily meal timing [11,19], while other studies reported no effect on EE [8,9,20]. Depending upon the particular study, changes were either observed in glucose tolerance and insulin resistance (in humans [10,11,19] and mice [17]), or were not observed (in humans [9] or mice [1]). These discrepancies in results of daily meal timing among studies of humans and between mice and humans have impaired definitive conclusions on the effects of daily/circadian meal timing.

However, there are clear differences in TRF paradigms used in humans versus mice that may elucidate the difference in circadian meal timing effects between the two species. While both human versus mouse studies have used a variety of protocols, generally the studies with humans modestly restrict feeding to only the active (day) interval [8–11,19,20], whereas mouse TRF regimens usually enforce the mice to feed only in their inactive (day) or active (night) intervals and/or have extended fasting intervals of up to 18 h [1,13,17,21]. Moreover, prior TRF protocols have not carefully deconvoluted the contribution of the *timing* within the circadian/daily cycle of the feeding episode from the *duration* of the *feeding* or *fasting*; in most prior protocols, both are changing, but which is most important has not been defined [9–11,19]. The daily timing of the eating interval has not been varied in a systemic way that allows comparisons between the mouse and human TRF studies. Another important issue that relates to the ‘real world’ of humans as opposed to laboratory studies is that prior laboratory TRF regimens for both mice and humans dictate complete fasting in the restricted intervals, but outside of the laboratory, humans usually have constant access to food. It is possible that involuntary fasting in the laboratory studies minimizes weight gain in a manner that is irrelevant to humans in their everyday life, who can snack whenever they wish.

The data of the Arble *et al*. study [13] suggest that a sudden increase in body weight occurs in the first one to two weeks of the TRF feeding regimen (electronic supplementary material, figure S1). This discontinuous shift appears to set the two groups of mice (i.e. day-fed versus night-fed groups) on different weight gain trajectories; after this shift in the first one to two weeks, the rate of weight gain is not very different between the two groups. This observation led us to question if the day-fed mice's metabolism was acutely shocked by the sudden change in daily feeding pattern from the preferred nocturnal feeding to a forced day pattern, but it subsequently adapts gradually to the altered feeding pattern after one to two weeks. Given that fasting in rodents can evoke a corticosterone (stress) response [22,23] and altered corticosterone levels are associated with metabolism and weight gain/loss [24,25], this sudden feeding shock might elicit a corticosterone response that could contribute to the effects of TRFs. Moreover, a comparison of the Arble *et al.* [13] TRF protocol (12 h day or night feeding coupled with a 12 h fast) with the Hatori *et al.* [1] TRF protocol (8 h night feeding, 16 h fasting) leads to another critical question, namely: is the dominant parameter for moderating weight gain the duration of the fast, or the timing of the allowed feeding window? Because the ‘conventional wisdom’ is that several hours of complete fasting at night is necessary to turn on ‘fat burning’ [5], this is a salient unresolved issue.

In this study, we used mice to compare the metabolic effects of differential timing of the HFD feeding window with or without a fast. Based on the fact that the circadian clock rhythmically modulates metabolism [5], we hypothesize that there is a daily phase for feeding that optimally regulates weight gain even in the absence of enforced fasting. In addition, we test whether the sudden switch from *ad libitum* food access to TRF initiates an interval of time during which metabolism adapts to the TRF, and moreover whether a corticosterone (stress) response is evoked. To perform these tests, we introduce a novel time-optimized feeding (TOF) regimen that separates the effects of phase-dependent feeding from the effects of enforced fasting in mice. Our data indicate that—while fasting has a significant effect—the daily timing of feeding is the predominant determinant of weight gain through differential carbohydrate/lipid oxidation. When the daily timing of nutrient intake (i.e. feeding) is temporally coupled tightly to circadian control of metabolic pathways, nutrient oxidation is more efficient, resulting in less lipid accretion. When feeding occurs at non-optimal times, there is a dramatic increase in cycle-to-cycle variability of carbohydrate/lipid oxidation as the animals' metabolism desperately searches for a favourable circadian metabolism linkage. This study highlights how the timing of feeding can influence weight gain independently of enforced fasting and provides a laboratory TOF protocol that is highly relevant to the feeding patterns of humans who usually have 24 h access to food.

## 2. Results

### 2.1. Novel TOF regimens to assess the need for enforced fasting

As mentioned in the Introduction, our examination of the weight gain data of the Arble *et al*. [13] study (electronic supplementary material, figure S1) led to the question whether the day-fed mice's metabolism was acutely shocked by the sudden change in daily feeding pattern from ad libitum feeding to forced TRF, but thereafter the mice's metabolism gradually adapts to the altered feeding pattern over the subsequent one to two weeks. We tested this hypothesis by continuously monitoring VO_2_ and VCO_2_ using indirect calorimetry as the mice transition to TRF using the feeding/fasting regimens introduced by Arble *et al*. [13]. The data shown in electronic supplementary material, figure S2 are of RER (respiratory exchange ratio, i.e. the ratio between the amount of CO_2_ produced in metabolism and O_2_ consumed, aka respiratory quotient), which is sensitive to changes in substrate oxidation. During the pre-treatment RC-AL interval, the typical pattern for RC-fed mice was observed. RER is high (indicating predominantly carbohydrate oxidation, CO) during the active (night) phase, while RER is low (predominantly lipid oxidation, LO) during the inactive (day) phase. After transfer to the experimental regimens, the group in which the HFD is available during the entire 12 h preferred nocturnal feeding phase (active/night; HF-night) maintains the same phase relationship of high RER at night (electronic supplementary material, figure S2A). However, the transfer of mice to HFD access for 12 h only during the non-preferred time during the light phase (day; HF-day) causes a sudden and dramatic shift in the daily rhythm so that high RER values shift to the day (when HFD is present for 12 h in the day; electronic supplementary material, figure S2B), indicating a temporal disruption of metabolic timing. This disruption reflects a change in the timing of food intake and subsequent oxidation. Nevertheless, over the next two weeks, the daily pattern of the RER rhythm partially adapts in the HF-day group, transitioning to a bimodal pattern when the mice are eating at dawn and dusk to accommodate as well as possible their preferred nocturnal eating pattern to a schedule when the food is available only in the light phase (electronic supplementary material, figures S2A and S2B). Interestingly, the amplitude of the RER rhythm increases initially in both the HF-night and HF-day groups in response to the altered regimens, but then this parameter also adapts after several days as it reverts to the pre-treatment amplitude. Therefore, the metabolism of the HF-day mice adapts partially but not completely to the altered feeding pattern by the end of the second week of TRF.

**Figure 1.**
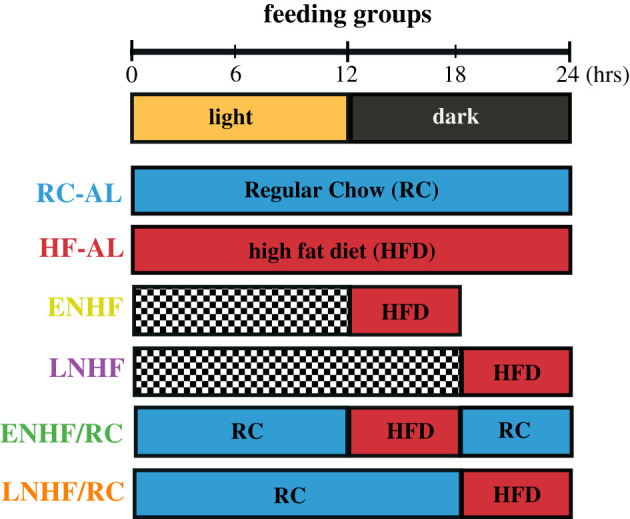
Design of novel feeding regimens. Mice were given regular chow ad libitum (RC-AL, blue) or HF-AL (red) as controls under a no feeding restriction protocol. To observe metabolic effects due to timing of feeding and fasting, mice were given HFD either the first 6 h of the dark cycle, aka LDT 12–18 (ENHF, yellow) or the last 6 h of the dark cycle, aka LDT 18–24 (LNHF, purple). To measure effects caused solely by feeding window, mice were given early or late night HFD access but were given ad libitum access to regular chow (ENHF/RC = green, and LNHF/RC = orange). Therefore, in the ENHF/RC and LNHF/RC groups, RC was present throughout even though the figure shows only 18 h of RC access. ENHF/RC is the key TOF group. All groups were in a light/dark cycle of 12 h of light and 12 h of dark (LD 12 : 12).

However, the regimens of Arble *et al*. [13] (and also of Hatori *et al.* [1]) do not disentangle the contributions of the timing of the feeding window from the duration/timing of fasting. To further refine these relationships, we designed a novel feeding paradigm to compare enforced fasting with RC ad libitum access (figure 1). Under these feeding regimens, mice were given a 6 h access to HFD during the night (active) phase of the 24 h cycle (LD 12 : 12), and this HFD access was either coupled with an enforced 18 h fast, or the enforced fast was eliminated by allowing 24 h ad libitum access to RC. HFD access was provided either during the first 6 h of the night (early night HFD (6 h) combined with complete fasting (18 h); hereafter ENHF), or during the second 6 h of the night (late night HFD (6 h) combined with complete fasting (18 h); hereafter LNHF). As mice are nocturnally active in LD 12 : 12, these windows of HFD availability correspond to either the first half (ENHF) or the second half (LNHF) of their active phase. Mice on the ENHF and LNHF regimens experienced the enforced 18 h fast every 24 h cycle. Mice on these feeding/fasting regimes were compared with mice with access to HFD at the same nocturnal phases but that had RC ad libitum (ENHF/RC), and therefore did not experience enforced fasting. We term these novel regimens ‘TOF’ regimens, in which the ENHF/RC regimen is particularly favourable. Because mice prefer to eat HFD if available, most of the calories consumed in the ENHF/RC and LNHF/RC groups occur during the EN and LN windows (see below). By comparing weight gain among these groups, we were able to distinguish effects due to an 18 h fast (e.g. ENHF versus ENHF/RC) versus effects due to shifting the daily phase of the feeding window in which the majority of calories were consumed (e.g. ENHF/RC versus LNHF/RC).

### 2.2. Weight gain under novel ‘time-optimized feeding’ regimens

**Figure 2.**
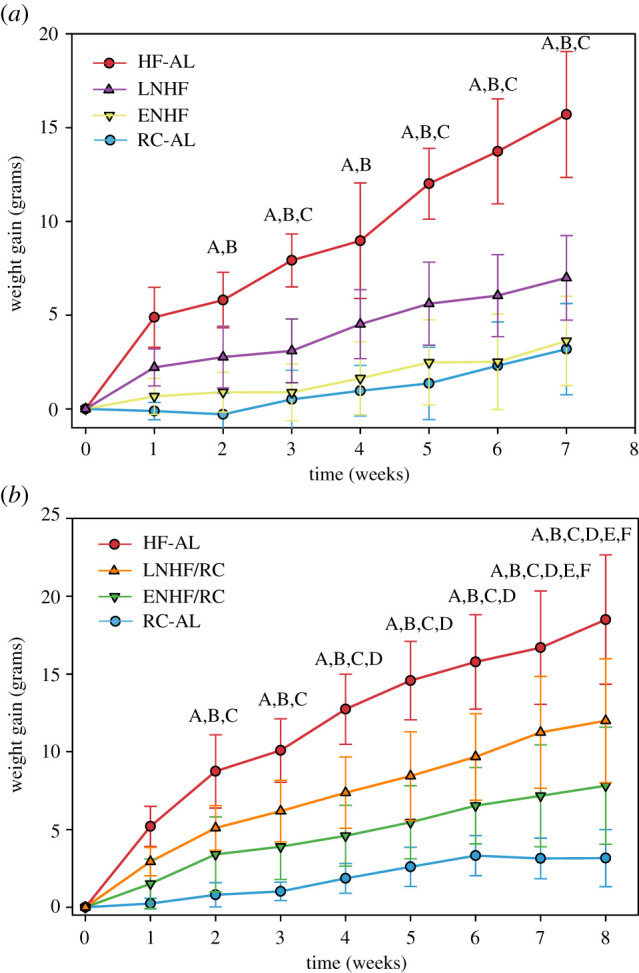
TRF of HFD causes weight gain in mice that is affected by allowing ad libitum access to regular chow. (*a*) TRF regimens. ENHF and LNHF without RC ad libitum: weight gain of mice given RC-AL (blue), HF-AL (red), ENHF (yellow) or LNHF (purple) over seven weeks (*n* = 7–10). ENHF and LNHF groups experienced an 18 h fast. Error bars indicate standard deviation. Letters indicate a significant effect (*p* < 0.05) after two-way repeated measures ANOVA analysis as follows: A = HF-AL versus RC-AL, B = HF-AL versus ENHF, C = HF-AL versus LNHF. These data are plotted as body weight rather than weight gain in electronic supplementary material figure S3A. (*b*) TOF regimens. ENHF and LNHF with RC ad libitum: weight gain of mice given RC-AL (blue), HF-AL (red), ENHF/RC (green) or LNHF/RC (orange) over eight weeks (*n* = 7–10). None of these groups experience an enforced fast. Error bars indicate standard deviation. Letters indicate a significant effect (*p* < 0.05) after two-way repeated measures ANOVA analysis as follows: A = RC-AL versus HF-AL, B = RC-AL versus LNHF/RC, C = HF-AL versus ENHF/RC, D = HF-AL versus LNHF/RC, E = RC-AL versus ENHF/RC, F = LNHF/RC versus ENHF/RC. These data are plotted as body weight rather than weight gain in electronic supplementary material figure S3B.

We compared weight gain effects of the TRF/TOF mice with or without RC *ad libitum*. Figure 2*a* shows weight gain over time for mice under RC-AL, HF-AL, ENHF and LNHF regimens. Mice on HF-AL have the largest increase in weight gain and mice on RC-AL maintain body weight similar to that at the start of the experiment, as expected from previous studies [1,17]. Mice that experienced an 18 h daily fast (ENHF or LNHF) gained less weight than did mice on HF-AL. When analysed using a two-way repeated-measures ANOVA, ‘feeding group’ and ‘weeks on feeding regime’ had a significant interaction (*p*-value < 0.001) with ‘body weight’ (electronic supplementary material, table S1A; see also electronic supplementary material, table S1B). Analysis of the calories consumed by the mice during the feeding regime showed the HF-AL group ate more calories than the other feeding groups, but there was no difference in chow consumed between the ENHF and LNHF groups, or between the ENHF/LNHF groups and RC-AL (electronic supplementary material, table S1C; see also electronic supplementary material, tables S1D–E). Further analysis using a Holms–Sidak post hoc test showed that over time, the HF-AL group gained significantly more weight than the RC-AL, ENHF or LNHF groups. However, the weights of mice on the ENHF and LNHF regimens were not significantly different over the course of the study (*p* = 0.075), suggesting both feeding regimes have a similar impact on weight gain.

Since the ENHF versus LNHF groups were not statistically different at the *p* < 0.05 level, we wondered if the 18 h fast—irrespective of the *timing* of feeding or fasting—was the primary cause of the weight gain differences between HF-AL versus ENHF and LNHF. Therefore, we performed another long-term weight gain experiment comparing RC-AL, HF-AL, ENHF and LNHF but this time, the EN- and LN-fed groups were also allowed access to RC ad libitum (therefore ENHF/RC and LNHF/RC), thereby circumventing any enforced fasting. As mentioned in the Introduction, TOF corresponds much more closely to the feeding behaviour of humans, who may or may not have regular meal times during their active intervals (daytime), but typically have 24 h access to food that allows snacking at any time [26].

We first measured long-term caloric intake and weight gain of mice under this non-fasting paradigm. We found mice in the three groups given HFD ate slightly more calories than the RC-AL group (electronic supplementary material, table S2D, *p*-values of 0.045–0.051; see also electronic supplementary material, table S2A–E). However, we saw no difference in caloric intake between the ENHF/RC and the LNHF/RC group or between the ENHF/RC and LNHF/RC groups and the HF-AL group (electronic supplementary material, table S2D, *p*-value = 0.993–0.913), similarly to what we found with the ENHF and LNHF feeding groups from the previous experiment (figure 2*a*; electronic supplementary material, table S1D). Despite the lack of difference in caloric intake, there were dramatic differences in the weight gain patterns among the various regimens (figure 2*b*; electronic supplementary material, table S2A). As before, HF-AL mice gained the most weight while RC-AL mice gained the least, however the new result is that all four feeding regimen groups were significantly different from each other in terms of weight gain (two-way ANOVA showed interaction between ‘weeks on regimen’ and ‘feeding group’ as *p*-value < 0.001). By the end of the 8-week study, there were significant differences between all groups; RC-AL mice showed little to no weight gain, HF-AL mice showed the largest increase in weight, and both the ENHF/RC and LNHF/RC mice exhibited intermediate weight gain (ANOVA corrected by the Holms–Sidak post hoc test). Importantly, the ENHF/RC and LNHF/RC groups were also significantly different from each other by the end of week 8 (figure 2*b*), with the ENHF/RC mice showing less weight gain than their LNHF/RC counterparts (*p* < 0.001). Therefore, this analysis of long-term weight gain demonstrates that the phase of the preferred feeding window (driven by the phasing of access to preferred HFD) affects weight gain even when forced fasting is eliminated. The difference between ENHF and ENHF/RC is significant (endpoint analysis showed *p* = 0.0279 when ENHF versus ENHF/RC are compared, see electronic supplementary material, table S7), but quantitatively, 79% of the benefit of the early feeding regime to the weight gain trajectory is attained even without fasting. Therefore, while an enforced fast minimizes weight gain, most of the advantage of the TRF regimen over HF-AL feeding is achieved without an imposed fast (e.g. ENHF versus ENHF/RC).

### 2.3. Metabolism under novel feeding regimens assessed by indirect calorimetry

**Figure 3.**
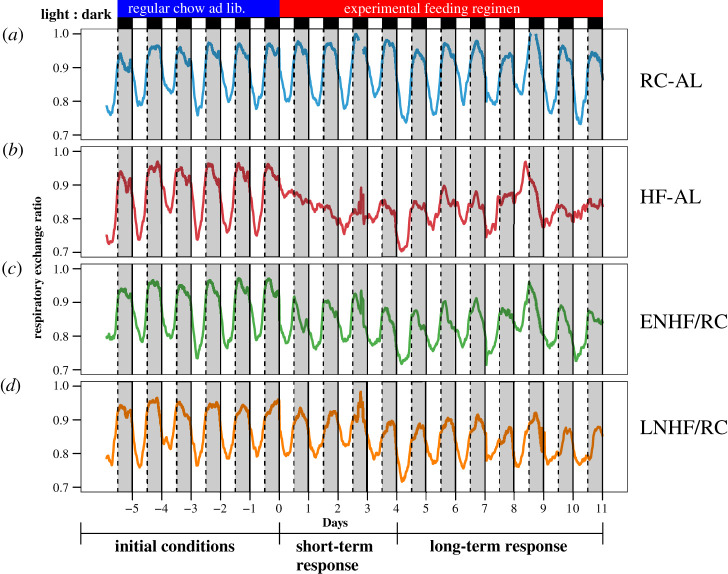
Indirect calorimetric measurements of RER rhythms of mice transferred from RC-AL to ENHF/RC, LNHF/RC, and HF-AL on day 0. Lines connect mean values for all mice in each group (*n* = 4 for each group). The total average kcal consumed by each mouse summed over days 0–9 were for the following groups: RC-AL: 102.4 kcal ± 20.5; HF-AL: 188.4 ± 24.1; ENHF/RC: 132.1 ± 5.3; LNHF/RC: 121.7 ± 12.0 (means ± s.d.). (*a*) RC-AL (RC ad libitum) throughout the experiment. (*b*) HF-AL , starting on day 0. (*c*) ENHF/RC (6 h HFD during LDT 12–18, RC ad libitum), starting on day 0. (*d*) LNHF/RC (6 h HFD during LDT 18–24, RC ad libitum), starting on day 0. Data are separated into temporal blocks: initial conditions (aka ‘baseline’) of regimen RC-AL for the 5.5 days prior to transfer to feeding groups, short-term response for days 0–4, and long-term response for days 4–11. Along the top of the figure, the blue horizontal bar represents the initial conditions (regular chow ad libitum), and the red horizontal bar indicates the ‘experimental feeding regimen’ beginning on day 0. Immediately below these blue/red bars, the white/black bars indicate the LD 12 : 12 light/dark cycle (day = white, night = black and grey-shaded areas).

To more fully interpret the metabolic processes underlying the differences among these feeding regimen groups, we studied mouse feeding, activity, and metabolism under our novel feeding regimens (figure 1) in specialized metabolic calorimetry chambers at the Vanderbilt Mouse Metabolic Phenotyping Center (MMPC). All mice were first on RC-AL for 6 days in the chambers, then put on one of four feeding regimens for 11 days (figure 3). During the pre-treatment RC-AL interval (aka ‘initial conditions’), RER is high during the active (night) phase, while RER is low during the inactive (day) phase. After transfer to the experimental regimens, there are important changes in the metabolic patterns among the groups in RER, CO and LO in the first four days (‘short-term response’ figure 3, table 1, and electronic supplementary material, table S4) which gradually adapt after 4 days so that the groups become more similar (‘long-term response’, figure 3; and electronic supplementary material, table S5). Consistent with our prediction, therefore the daily phase of the HFD feeding window significantly affects daily metabolic patterns (assayed here by indirect calorimetry) even in the absence of enforced fasting.

**Table 1.** Summary of treatment × day mixed-model analysis; *p*-values for interactions between treatment and day for all four regimens.

metric assessed	initial conditions	short-term response	long-term response
RER	0.45	0.001	0.099
carb. ox.	0.576	0.001	0.115
lipid ox.	0.651	0.001	0.195
activity	0.945	0.917	0.092
kcal consumed	0.906	0.118	0.249
kcal burned (EE)	0.953	0.264	0.012

During the first 6 d of the experiment (all mice on RC-AL), there were no differences among the groups in kcals consumed or kcals burned (table 1; electronic supplementary material, tables S3E/F and 5E/F) and strong daily RER rhythms persisted with high values during the active (nocturnal) interval, indicating higher CO in the night (figure 3). Following implementation of the experimental feeding regimens, HF-AL and LNHF/RC mice showed a slight increase in kcals consumed compared with the RC-AL group only (electronic supplementary material, table S5E). There were some differences in ‘kcals oxidized’ (‘kcals burned’, aka energy expenditure, EE) with the HF-AL group eating more calories than the RC-AL group but occasionally the HF-AL group also ate more than the ENHF/RC and LNHF/RC groups (table 1; see electronic supplementary material, tables S5F for detailed analyses). Importantly, there were no significant differences in daily caloric intake between the key ENHF/RC and the LNHF/RC groups (table 2; electronic supplementary material, table S6). Finally, locomotor activity levels and phasing were equivalent among all groups. While we see a peak in activity in the LNHF/RC group at LDT18 (light/dark time (aka ZT), where LDT0 and LDT24 = dawn of a LD 12 : 12 cycle and LDT12 = dusk of a LD 12 : 12 cycle) that suggests a food anticipatory response, this activity did not significantly impact overall daily activity (tables 1 and 2; electronic supplementary material, figure S4). There are differences in weight gain between the ENHF/RC and LNHF/RC groups in the long term (figure 2*b*), but we found no significant difference in EE or kcals consumed between these two groups during the first ten days on their respective feeding regimes (table 2; see the mixed-model analyses in electronic supplementary material, tables S3E/F, S4E/F, S5E/F and S6E/F). As described below, the weight gain of mice on each regimen is correlated between the timing of feeding and the timing/variability of nutrient oxidation, not with the difference between kcal consumed and kcal burned {(kcal consumed) − (kcal burned) = energy balance}. Notably, there were no significant differences in daily caloric intake or EE between the ENHF/RC and the LNHF/RC groups (table 2).

**Table 2.** Summary of treatment × day mixed-model analysis; *p*-values for interactions between treatment and day for ENHF/RC versus LNHF/RC only.

metric assessed	initial conditions	short-term response	long-term response
RER	0.331	0.615	0.112
carb. ox.	0.306	0.704	0.222
lipid ox.	0.615	0.309	0.235
activity	0.920	0.750	0.070
kcals consumed	0.921	0.479	0.465
kcals burned (EE)	0.567	0.575	0.580

**Figure 4.**
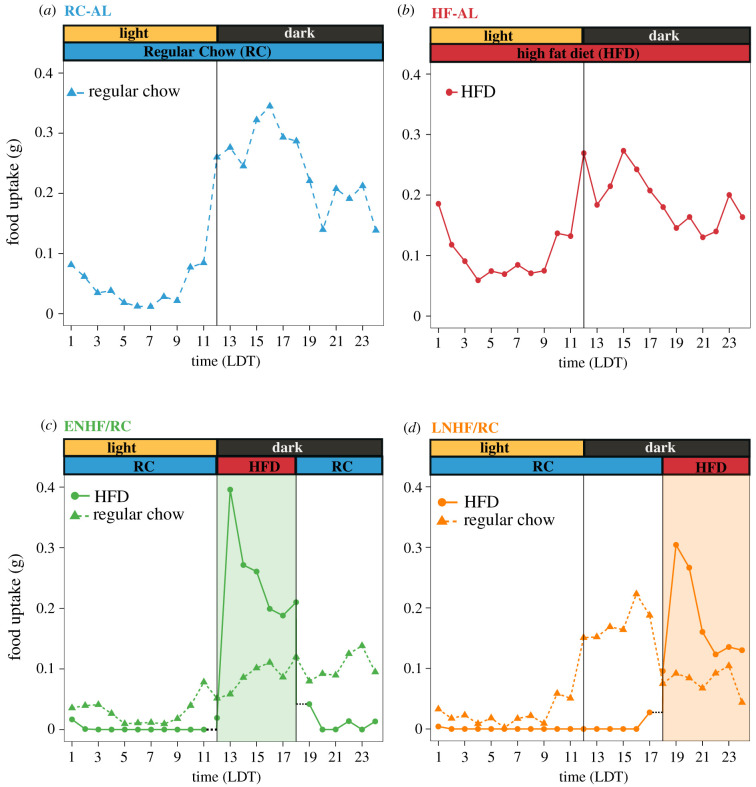
Mice with phase-restricted access to HFD and ad libitum access to RC eat throughout the active nocturnal phase. Feeding profiles for RC-AL (*a*), HF-AL (*b*), ENHF/RC (*c*) and LNHFD/RC (*d*) mice. Average hourly food intake for HFD (dots) and RC (triangles) is plotted for each feeding group based on the full 10 d feeding regime. For ENHF/RC and LNHF/RC, the shaded regions indicate when HFD was available for mice in that group.

To confirm that mice on the non-fasting HFD TRF regimens (ENHF/RC and LNHF/RC) were not undergoing a self-imposed fast when they had access to only RC, we monitored the timing and rate of food uptake throughout the study. Figure 4 shows the average hourly food intake over 24 h for each of the four feeding groups used in the calorimetry chambers (these 24 h plots are the average values over the full 10 d of the feeding regimens). While RC-AL and HF-AL groups were allowed unrestricted 24/7 access to food, they primarily fed in the nocturnal interval when their locomotor activity was high (electronic supplementary material, figure S4) during the 12 h of darkness (especially early in their night; figure 4*a,b*), but they did not voluntarily fast completely during their daytime phase. The HF-AL group consumed more food during the light portion as compared with the RC-AL group, consistent with previous literature showing that mice develop less rhythmic feeding patterns when given HF-AL [27,28]. For the mice under either ENHF/RC or LNHF/RC, the shaded region indicates when mice were allowed to eat HFD (figure 4*c,d*). We found that for both regimens, mice prefer to eat throughout the 12 h dark period in a manner similar to the RC-AL group, supplementing their diet with RC when HFD was not available. The majority of their calories came from the HFD, as HFD was preferred when it was available; nevertheless, both ENHF/RC and LNHF/RC mice received 25–35% of the kcals in their diet from RC.

### 2.4. Corticosterone stress responses are not evoked by TRF regimens

The abrupt shift in the weight-gain data of Arble *et al*. [13] and our observations of the short-term response in RER values (figure 3, table 1; electronic supplementary material, table S4) suggested that the sudden change in daily feeding pattern from ad libitum feeding to forced TRF might have evoked a stress involving corticosterone. Corticosterone is a critical hormone that regulates metabolism and weight. Release of this glucocorticoid from the adrenal cortex signals glucose secretion into the blood stream and a decrease in fatty acid oxidation [29]. Some studies of fasting in rodents have reported an elevated corticosterone response [22,23] and adrenalectomy is sometimes associated with changes in food intake and weight [24,25]. Moreover, corticosterone levels exhibit a daily rhythm in mammals, with peak values near the onset of activity and troughs at sleep initiation [30]. Because mice might perceive the enforced 18 h fast as a stress, we reasoned that it might evoke a corticosterone response. Also, extra corticosterone release could occur as an anticipatory peak which is sometimes observed when food access is limited to only a few hours. By either interpretation, an enhanced corticosterone response could promote metabolic consequences including weight gain/loss [23,24]. We therefore hypothesized that TRF regimens [1,13] might elevate corticosterone levels. This prediction, which has not been previously tested, can be evaluated with our novel TOF feeding regimen protocols.

Therefore, we compared corticosterone levels in mice undergoing our regimens: RC-AL, HF-AL, ENHF, LNHF, ENHF/RC and LNHF/RC, with the goal of determining if changes in the level of corticosterone in mice under TRF protocols correlate with the weight gain profiles shown in figure 2. To assess what time of day would be the best for comparing corticosterone levels among the regimens, we first confirmed that the daily pattern of corticosterone was consistent among the regimens, and indeed the patterns were the same with peaks at LDT12 and troughs at LDT0/24 (electronic supplementary material, figure S5). Based on our data and those of other researchers that indicate the LDT12 peak level of corticosterone is most variable and most sensitive to perturbations [31–33], we selected this daily phase for our comparisons. We then compared the values of corticosterone for the various feeding regimens on days 0, 3, 7 and 14 to test (i) if any of the regimens—including ± fasting—evoke a corticosterone response, and (ii) if there is a time-dependency/adaptation period after transfer to the different regimens with regard to corticosterone levels (electronic supplementary material, figure S6). We found no consistent or significant time-dependency differences among the groups for days 3, 7 or 14 after the regimens were initiated (electronic supplementary material, figure S6B), nor were there any overall differences in corticosterone levels among the groups (electronic supplementary material, figure S6C; there is a small difference between the ENHF group and the RC-AL/HF-AL groups, but the other three groups were not different). In particular, the data show that neither fasting nor presenting the HF at different circadian/daily times leads to a consistently elevated corticosterone level, and therefore that the differences in EE/weight gain among the TRF protocols are not associated with a corticosterone reaction. This result confirms the conclusions of other studies in mice and rats, where HF-AL can depress the amplitude of the daily corticosterone rhythm, but various TRF regimes did not evoke a corticosterone response [22,31–33].

### 2.5. Lipid and carbohydrate oxidation under novel feeding regimens

**Figure 5.**
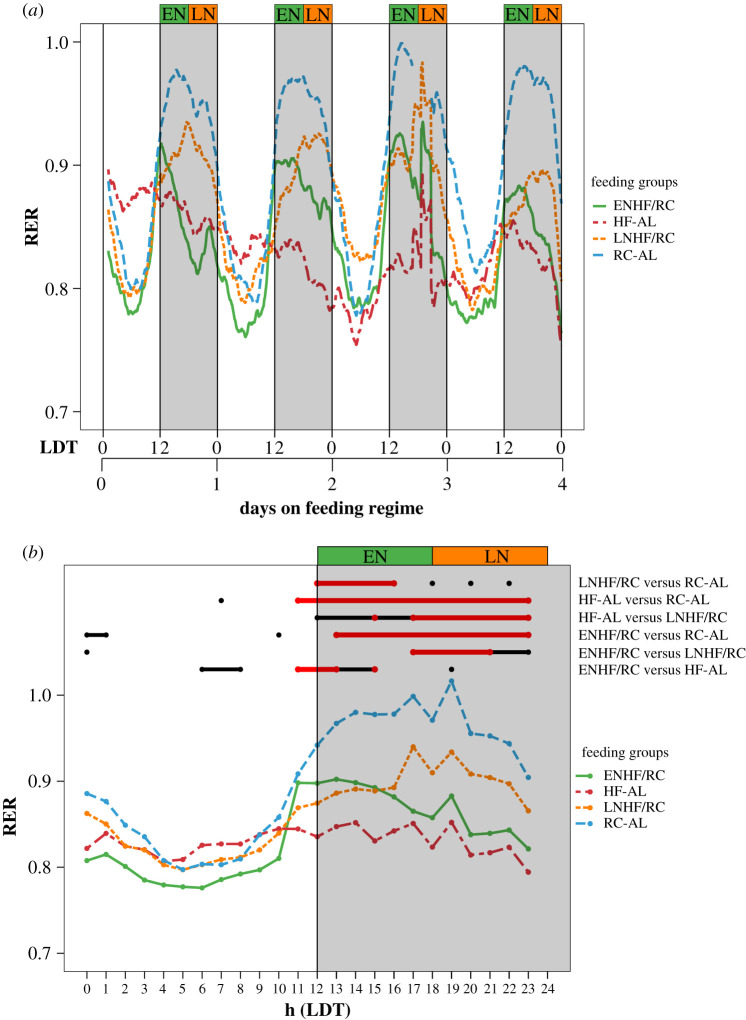
The daily timing of HFD consumption affects the phase of RER rhythms and ad libitum access to HFD decreases daily RER values in days 0–4. Lines connect mean values for all mice in each group (*n* = 4 for each group). (*a*) Average RER for each feeding group: HF-AL (red), ENHF/RC (green), LNHF/RC (orange) and RC-AL (blue) during days 0–4 on feeding regimes. Shaded regions indicate the 12 h night (lights-off). (*b*) From the mixed-model analysis for days 0–4 (electronic supplementary material, table S4A), the daily phases for which RER values are significantly different (*p* < 0.05 in black, *p* < 0.01 in red) among the groups are shown. The average RER values for each hour from days 0 to 4 are represented in a single 24 h format.

Because the shift to TRF regimens did not stimulate a corticosterone response, we reasoned that the sudden transfer caused a disruption in metabolic switching of substrate preference (CO versus LO) over the daily cycle. Support for that hypothesis came from the large difference among the regimens in terms of RER (figures 3 and 5) coupled with the absence of significant differences among the groups in terms of kcals consumed (tables 1 and 2). A mixed-model analysis conducted on RER data comparing all four regimens showed a significant interaction between ‘days on feeding regime’ and ‘regimen treatment’ on RER in days 0–4 (*p* = 0.001, ‘short-term response’ in table 1; electronic supplementary material table S4A). We performed another mixed-model analysis on the initial 6 d when all mice were on RC-AL (initial conditions) and found no significant difference, suggesting this effect did not come from an inherent bias among treatment groups prior to the change in their feeding regime (*p* = 0.45, ‘baseline’ in electronic supplementary material, table S3). These statistical differences can be attributed to two important differences in the RER patterns among the groups. First, all three groups with access to HFD had a lower average RER, as expected since animals with access to HFD eat more fat, and the resulting increase in lipid metabolism will lower the RER values (figure 5*a,b*). Second, the peak phase of the RER rhythm was shifted so that there were different phase relationships between the RER rhythm and the LD cycle; RER is highest in both the HF-AL and ENHF/RC groups in the early portion of the dark interval, whereas the RER of the LNHF/RC group peaks in the latter half of the dark interval (and the peak of RER in RC-AL is roughly centered within the 12 h dark interval, figure 5). The impact of the feeding group on RER also appeared to be largest during the first few days on the new regimens (short-term response); after that, average and peak RER values rapidly returned to levels comparable to those of the RC-AL group (by as soon as day 8, figure 3).

**Figure 6.**
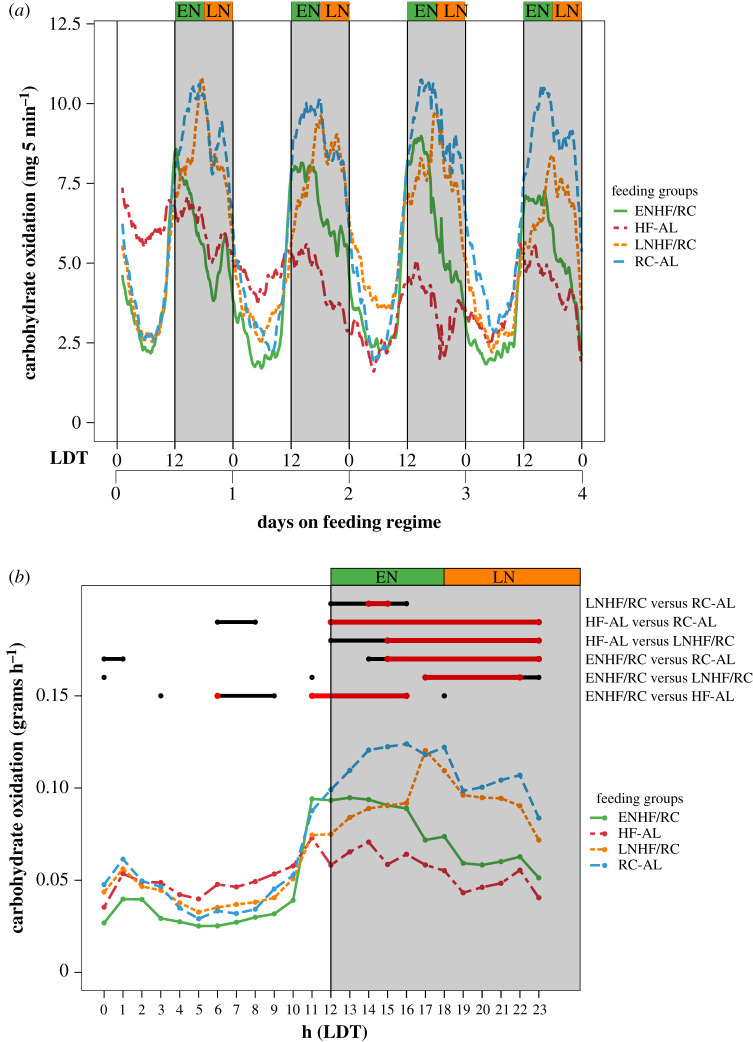
CO is dependent on time of HFD presentation in days 0–4. Lines connect mean values for all mice in each group (*n* = 4 for each group). (*a*) Average CO for each feeding group: HF-AL (red), ENHFD/RC (green), LNHF/RC (orange) and RC-AL (blue) during days 0–4 of the feeding regimes. Shaded regions indicate the 12 h night (lights-off). (*b*) From the mixed-model analysis for days 0–4 (electronic supplementary material, table S4D), the daily phases for which CO values are significantly different (*p* < 0.05 in black, *p* < 0.01 in red) among the groups are shown. The average CO values for each hour from days 0 to 4 are represented in a single 24 h format.

Changes in RER indicate altered oxidation of carbohydrates versus lipids, so we next calculated rates of CO and LO based on the RER data [34,35]. As with RER, we found for CO an interaction between ‘day’ and feeding regimen ‘treatment’. There was also a notable difference in the phase relationship of the CO peak relative to the night between the ENFH/RC and LNHF/RC such that maximum CO occurred at the times when HFD was presented (figure 6). Moreover, the largest differences in CO phase among the groups occurred in days 0–4 (‘short-term response’, figure 6*a*), and each group thereafter gradually converged on patterns that were more similar to the RC-AL group by day 8 and onward. For CO, the HFD-fed groups (HF-AL, ENHF/RC and LNHF/RC) were different from the RC-AL group in days 4–11 (long-term response), but the differences were notably smaller than for days 0–4 (compare electronic supplementary material, table S4D with table S5D).

**Figure 7.**
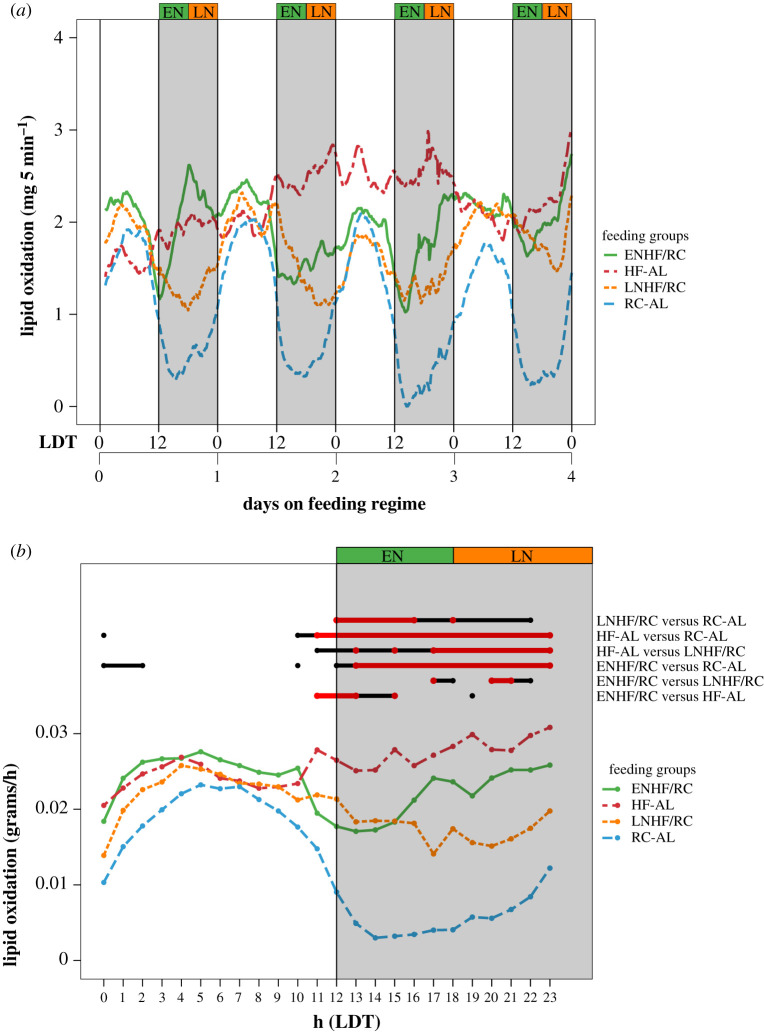
Lipid oxidation is dependent on time of HFD presentation in days 0–4. Lines connect mean values for all mice in each group (*n* = 4 for each group). (*a*) Average lipid oxidation (LO) for each feeding group: HF-AL (red), ENHF/RC (green), LNHF/RC (orange) and RC-AL (blue) during days 0–4 on feeding regimes. Shaded regions indicate the 12 h night (lights-off). (*b*) From the mixed-model analysis for days 0–4 (electronic supplementary material, table S4B), the daily phases for which CO values are significantly different (*p* < 0.05 in black, *p* < 0.01 in red) among the groups are shown. The average LO values for each hour from days 0 to 4 are represented in a single 24 h format.

Finally, we investigated the impact of feeding regime on lipid oxidation (LO). Once again, we found a significant interaction for LO between feeding regimen ‘treatment’ and ‘days’ (*p* < 0.001, table 1; electronic supplementary material, table S4B). Mice in the ENHF/RC and LNHF/RC groups experienced a dip in LO when HFD was present during the active night interval (figure 7). Interestingly, both the ENHF/RC and LNHF/RC groups began to increase LO towards the end of their respective access to HFD. However, given that the ENHF/RC group had HFD presented at an earlier time than the LNHF/RC group, this led to higher LO in the ENHF/RC group at the time of the transition to lights-on than in the LNHF/RC group (figure 7*a*). This effect is observed until day 4, then diminishes in the later days. Peak LO for all groups occurs in the middle of the day and is similar among the feeding groups. On average, LO was higher in the ENHF/RC group than in the LNHF/RC group and different from the HF-AL group by day 3 (figure 7*b*). These data indicate that the timing of the access to HFD within the nocturnal active phase is affecting the LO rate, with mice feeding early in the active phase burning more lipids during the 4 days after day 0 in which they are adapting to a new feeding regimen (short-term response).

### 2.6. Variability of nutrient oxidation profiles reveals metabolic instability

**Figure 8.**
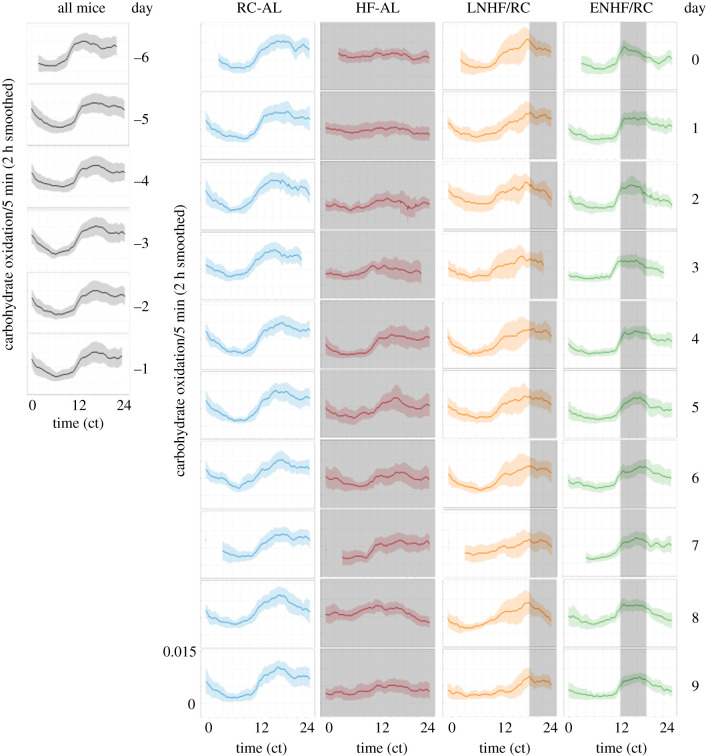
Day-by-day CO patterns and variation among the four regimens. Data are the CO rate per 5 min as a function of time of day (ct = circadian time), presented as a 2 h smoothing average of CO with an envelope of ± 1 s.d. among the animals in each group. The first 6 days (days −6 to −1) are all mice in one group on the RC-AL regimen (grey curves and envelopes). On day 0, the mice were split into four groups and monitored for 10 more days (days 0–9): (1) animals maintained on RC-AL (blue curves and envelopes), (2) ENHF/RC (green curves and envelopes), (3) LNHF/RC (amber curves and envelopes), and (4) HF-AL (red curves and envelopes). Times of access to HF diet are shown as translucent grey boxes. Missing data on days 0, 3 and 7 are due to cage changes and rebooting of the data acquisition system.

**Figure 9.**
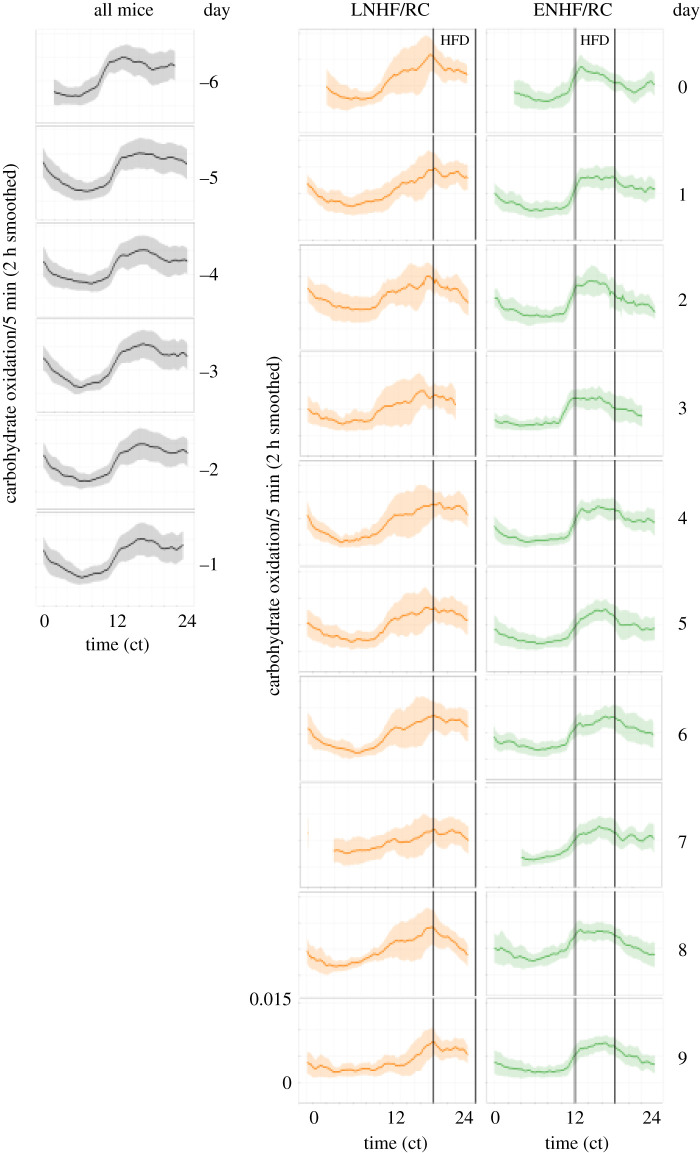
Direct comparison of CO patterns and variation between ENHF/RC and LNHF/RC. Data are plotted as in figure 8, except comparing only the ENHF/RC and LNHF/RC groups after transfer to the novel feeding regimens (days 0–9). Variation of CO among the animals in each group is shown as an envelope of ± one standard deviation. ENHF/RC is green curves and envelopes, while LNHF/RC is amber curves and envelopes. Missing data on days 0, 3 and 7 are due to cage changes and rebooting of the data acquisition system.

Further analysis of these data indicated that the variability of metabolic responses between animals and cycles among the four groups was distinctly different; in particular, the variability of the LNHF/RC group data was much higher than in the other three groups. This effect was true for both CO and LO, and the CO data are shown in figures 8 and 9. Before transfer to the feeding regimens (days −6 to −1), the daily CO patterns exhibited low variability with higher values during the early-night phase of preferred feeding (figure 8). This temporal profile and low variability continued in the RC-AL and ENHF/RC groups after transfer to the altered feeding regimen for ENHF/RC (days 0–9). As also shown in figure 6, the daily rhythm of CO flattened out in the HF-AL group after transfer on day 0 and the variability among cycles and animals remained low (figure 8). In striking contrast, however, the LNHF/RC dramatically increased in cycle-to-cycle and inter-individual variability after transfer on day 0; the variability was especially high in the early night when the time of preferred feeding (figure 4) is conflicting with the circadian regulation of nutrient oxidation (figures 6 and 7). When the variability data for the two key groups ENHF/RC versus LNHF/RC are explicitly compared, these differences become obvious (figure 9). Note also that while the average temporal patterns of CO and LO appear to adapt over time (long-term response is smaller than short-term response; table 1), the cycle-to-cycle variability from day 0 to day 9 persists throughout the experimental timecourse (figures 8 and 9). Therefore, in animals whose circadian rhythm of metabolism is coupled to nutrient intake tightly and therefore matches one another, the oxidation pathways coordinate with nutrient intake and therefore there is much more efficient nutrient oxidation and less lipid accretion. When the timing of metabolism and nutrient intake are mismatched, instability ensues—as indicated by increased variability—as metabolism searches for an optimal synergism that is ultimately unattainable.

## 3. Discussion

The relevance of daily eating patterns to health and disease is a very active area of research that includes both rodent and human studies [5,6]. Despite the growing number of TRF studies, however, it remains uncertain whether the beneficial effects of TRF are due to the eating/fasting duration or to the daily phasing of the eating/fasting window (or a combination of both) [1,6,9–11,17,20,26]. Interpretation of previous research is confounded by study designs that included an imposed fasting period. Because fasting itself has direct effects on metabolism, results based on TRF protocols that include an enforced fast (in some cases, also fasts of different duration) are not definitive in terms of understanding whether the timing of feeding is important. Moreover, extrapolating studies with mice that enforce an obligatory fast to humans who usually have 24 h access to food are of limited relevance.

Our novel TOF regimens disentangle these factors by specifying a preferred eating interval during the day/night cycle via access to HFD while avoiding fasting/stress with ad libitum access to RC. Mice given time-restricted HFD with RC ad libitum will feed mostly when HFD is available, but have snacks of RC when HFD is not available, and these RC snacks account for 25–35% of their daily caloric intake. The timing during the day/night cycle of the HFD presentation leads to differences in weight gain, with HFD presented at the onset of activity (ENHF) causing less weight gain than if presented later (LNHF, figure 2*a*). Unexpectedly, mice given ad libitum RC along with the time-restricted HFD (ENHF/RC and LNHF/RC) show similar weight gain trends as their counterparts that experienced 18 h fasts (ENHF and LNHF, compare figure 2*a* with figure 2*b*). Mice on ENHF/RC and LNHF/RC feed throughout the day, but they do not consume more calories than their fasting counterparts (ENHF and LNHF). Nevertheless, even in the non-fasting groups ENHF/RC and LNHF/RC, a large majority of calories are ingested during the 6 h HFD window. Therefore the observed differences in weight gain are mostly due to disruptions of the circadian metabolic regulation and not to a fasting response *per se*. Importantly, the timing of the 6 h HFD meal within the period of locomotor activity has a significant impact on weight gain, with mice eating the majority of calories in the first 6 h of the night (onset of activity; ENHF/RC) having lower weight gain than mice that consumed most of their calories in the latter half of their active phase (LNHF/RC, figure 2*b*).

To be clear, there *is* an effect of fasting when HFD is present in the early night versus late night. When an 18 h fast is present, the weight gain of the mice on the early-night HFD regimen is indistinguishable from those on chow ad libitum (RC-AL, figure 2*a*), but when chow is made available ad libitum in combination with HFD, ENHF/RC mice gain slightly more weight than the RC-AL group (figure 2*b*). However, compared with the RC-AL group, the weight gain of the LNHF/RC group is not different from that of the LNHF group (figure 2*a* versus 2*b*). Therefore, fasting reduced weight gain when HFD was present during the early nocturnal phase (approx. 20% of the total benefit of the early feeding regimens can be attributed to fasting; the early nocturnal phase is when mice are most active), but not when HFD was present in the second half of the night. Taken together, these data suggest that the major determinant of the impact on weight gain is *when* calories are eaten rather than the presence or the absence of fasting. Ingesting the majority of calories at the onset of the main activity period and metabolic demand limits weight gain. Snacking calorically dense meals out of phase with the peak metabolic demand causes a bias towards weight gain, and dramatically increases the cycle-to-cycle variability in temporal oxidation patterns (figures 8 and 9). We reached very similar conclusions in a study with humans and the daily timing of feeding [8].

**Figure 10.**
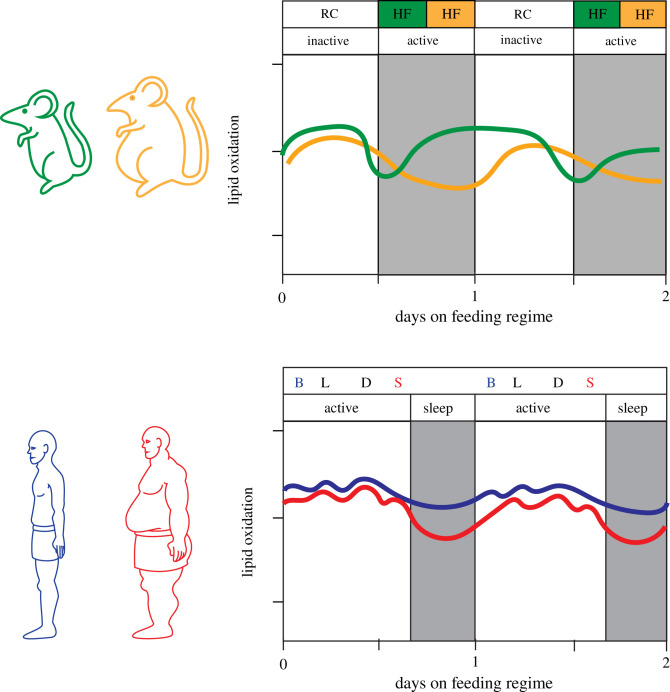
Timing matters: eating when activity levels are high enhances lipid oxidation, thereby reducing fat accumulation. (*a*) Mice: graphical representation of lipid oxidation under the ENHF/RC (green) and LNHF/RC (orange) during the short-term period (days 0–4 of feeding regime); this depiction is a redrawing of the data in figure 7*b*. ENHF/RC lipid oxidation dips earlier and shallower in the active (nocturnal) phase than LNHF/RC, followed by achieving max lipid oxidation during the inactive (diurnal) phase earlier and longer than mice under the LNHF/RC regime. This pattern results in more total lipids to be oxidized by the ENHF/RC mice than by the LNHF/RC mice. (*b*) Humans: graphical representation of lipid oxidation data in subjects given a breakfast, lunch and dinner regimen (BLD; blue) versus a lunch, dinner and snack regimen (LDS; red) [8]. Eating earlier in the active (diurnal) phase shows similar effects to lipid oxidation as in mice, with the BLD session burning more total lipids during the inactive (nocturnal) period than the LDS session (modified from [8]).

Our feeding/fasting regimens appear to not evoke a corticosterone response, so one of our initial hypotheses was not supported. However, the daily patterns of metabolic status were clearly modulated by our feeding/fasting protocols. To assess the metabolic state under the different regimens, we performed a three-week metabolic analysis of mice on shifted restricted/unrestricted feeding protocols using indirect calorimetry. EE, respiratory exchange ratio (RER), and oxidation of carbohydrates/lipids were thereby measured based on VO_2_ and VCO_2_ values; among the various groups, there are no significant differences in EE, daily caloric intake, or activity. We found that caloric intake and calories burned were not different between the ENHF/RC and LNHF/RC groups (table 2), which had been thought to be the primary mechanism for the resistance to weight gain of mice that experience fasts of varying duration [1,17]. Instead, we find that the effects on weight gain are due to changes in substrate oxidation, with the LNHF/RC group preferentially burning quantitatively more carbohydrates and fewer lipids as compared with the ENHF/RC group, causing a difference in weight gain (figures 6*b*, 7*b* and 10*a*). These results are similar to what our laboratory and other research groups have previously reported in humans, where the shifting of a breakfast meal to a late-evening snack led to altered lipid oxidation compared to when the subjects ate a breakfast, lunch, and dinner (figure 10*b*) [5–8,10,36–39]. From this study, we conclude that mice respond in a similar manner as humans did to temporal shifts in food consumption. Moreover, because of the temporal mismatch between preferred feeding time and the circadian CO/LO switch in the LNHF/RC group, metabolic stability is lost (higher variability in the LNHF/RC group, figures 8 and 9) so that nutrient oxidation becomes less efficient, resulting in more lipid accretion.

The effects of differential carbohydrate/lipid oxidation are temporary. In the first 4–5 d after transfer to different feeding/fasting regiments, we observed a transient disruption in RER caused by a shift in the timing of CO and LO (short-term response). This initial shift in the RER pattern indicates a preferential burning of carbohydrates by the LNHF/RC group in the late night that then extends into their inactive daytime phase. This phasing leads to fewer lipids oxidized overall, which was not observed from mice in the ENHF/RC group. Thus, when feeding occurs at non-optimal times—as in the LNFH/RC group—the animals' metabolism restlessly casts about for a favourable temporal linkage between food intake and metabolism, which dramatically increases the cycle-to-cycle variability of carbohydrate/lipid oxidation, perhaps with tissue-specific dynamics. After 4–5 d in the new protocols, mice partially adapt to their new schedules and exhibit more similar metabolic profiles over the daily cycle (however, the variability persists at least until day 9; figures 8 and 9). This suggests that the long-term weight gain effects seen in our mice are due to short-term carbohydrate/lipid oxidation imbalances and variability as the mice adapt to their new feeding times. Indeed, by the conclusion of the indirect calorimetry study, the fat mass and body fat percentage were already altered in the manner we expected from the long-term weight gain study (electronic supplementary material, figure S7). In particular, after only 10 d on the new feeding regime (before differences in weight gain were detectable), ENHF/RC and LNHF/RC already have a lower body fat percentage than the HF-AL group, and ENHF/RC have a lower average LNHF/RC body fat mass (electronic supplementary material, figure S7). In our study, the largest increases in weight gain occur during the first week, which set the groups on different weight-gain trajectories that ultimately lead to significant differences in body weight after a period of several weeks (figure 2*b*). The data of other studies appear to follow this temporal pattern as well [1,13,17,31].

Our data suggest that weight gain by mice in TRF regimens is predominantly determined by the *timing* of the daily eating interval (approx. 80% for the early night regimens), while the presence/absence of fasting is a significant but less important component (approx. 20% for the early night regimens). The temporal coupling and phasing of nutrient intake (eating behaviour) with circadian phasing of metabolism drives a CO/LO switch in substrate preference that determines weight gain without restricting food access. Optimal temporal coupling between the time of feeding and the time of the CO/LO switch results in stable metabolic adaptation. In addition, while there is certainly a transient adaptation interval of metabolism to TRF regimens, a corticosterone stress response is not triggered. These results highlight how the timing of ingestion of calorically dense foods as well as the presence/absence of an interval of fasting are important determinants of weight gain.

## 4. Material and methods

### 4.1. Treatment groups

Eleven-week-old male C57BL/6 J mice were individually housed under a light/dark cycle of 12 h light followed by 12 h dark (aka LD 12 : 12) at 22° ± 2°C. To separate the beneficial effects of a restricted feeding time versus a prolonged fasting period, we implemented the novel TOF regimens depicted in figure 1 [16]. After one or more weeks of acclimation to LD 12 : 12, the mice were placed in one of the following treatment groups: regular chow ad libitum (RC-AL), HFD and libitum (HF-AL), early night HFD with an 18 h fast (ENHF), late night HFD with an 18 h fast (LNHF), early night HFD with regular chow ad libitum (ENHF/RC), and late night HFD with regular chow ad libitum (LNHF/RC) (figure 1). ENHF/RC is the key TOF group. Early-night groups were given HFD during the first 6 h of the 12 h night and late-night groups were given HFD during the last 6 h of the 12 h night. Mice with RC-AL were provided with unlimited access to regular chow from a separate feeding container. Cages were examined daily at the transitions when HFD food access was switched on/off to ensure that no HFD chow was left in the cage or hoarded. Regular chow (RC) was purchased from LabDiet (Rodent 5001) and contains 13% calories from fat. High-fat diet (HFD) was purchased from Research Diets (D12492) and contains 60% calories from fat. All animal experiments were approved by the Vanderbilt University Institutional Animal Care and Use Committee and were conducted according to that committee's guidelines.

### 4.2. Measurements of indirect calorimetry

Twelve-week old male C57BL/6J mice (*n* = 4 for each group) were weighed, divided into groups, and allowed to entrain to a LD 12 : 12 cycle for one week. After entrainment, mice were individually housed in Sable Systems respiratory chambers at Vanderbilt University's Mouse Metabolic Phenotyping Center (MMPC) to monitor the rates of VCO_2_ and VO_2_ at 5-min intervals which were used to calculate the RER (as VCO_2_/VO_2_). EE was also calculated from the VCO_2_ and VO_2_ data using the Weir heat equation. CO and LO rates were calculated using equations described by Frayn, Hall and co-workers [34,35]. Mice were weighed weekly. HFD and RC were placed in automated feeders that recorded the quantity and time of chow retrieval. Mouse activity was monitored via infrared beam detectors. Body composition was determined for mice during day 6 and day 10 of feeding regimens using a Bruker Minispec Body Composition Analyzer.

### 4.3. Long-term weight-gain experiments

Twelve-week-old male C57BL/6 J mice (*n* = 7–10 per feeding group) were housed in a light-controlled box to maintain light/dark cycles. Prior to experiments, the singly housed mice were weighed, divided into groups, and allowed to entrain to a LD 12 : 12 cycle for one week. For seven weeks, mice were weighed every 7 d during the TRF regimen. For restricted HFD groups, high-fat chow was removed and added manually and cages were checked for hoarded pellets that were then removed. The amount of HFD and RC was weighed at the beginning of the light cycle each day.

### 4.4. Corticosterone experiments

For the test of daily patterns of corticosterone level, 11-week old mice were entrained to a LD 12 : 12 cycle for one week. Following entrainment, mice were put on one of the feeding regimens: RC-AL, HF-AL, ENHF, LNHF, ENHF/RC or LNHF/RC. Mice underwent these feeding protocols for 14 d. At the end of 14 d, the mice were sacrificed by cervical dislocation at LDT0, LDT6, LDT12, LDT18 and LDT24 (*n* = 5 for each collection time per feeding protocol) and had their trunk blood collected. To avoid an unwanted corticosterone elevation due to the stress of handling and euthanasia, mice were gently handled and sacrificed in under two minutes (mice were brought to the sacrifice room from the light control room by one ‘clean’ individual, and the sacrificing and tissue collection was performed by a different researcher). All mice were sacrificed ±30 min of the specified LDT time. For the test of corticosterone levels on days 0, 3, 7 and 14 of the feeding regimens, 11-week-old male C57BL/6J mice were entrained to LD 12 : 12 for one week, after which time they were transferred one of the feeding regimens. Mice were sacrificed at LDT 12 on days 0, 3, 7 and 14 of their respective regimens via cervical dislocation and their trunk blood was collected. For all experiments, trunk blood was immediately centrifuged to isolate and collect serum. Corticosterone levels in serum were measured using a commercially available corticosterone ELISA assay (ENZO Life Sciences).

### 4.5. Statistical analyses

For the long-term weight gain experiments (shown in figure 2), a two-way repeated measures ANOVA for interactions of feeding groups and time was performed using R and Sigmaplot. The Holms–Sidak post hoc test was used to correct for multiple comparisons. For the corticosterone experiments, statistical significance between groups was determined using two-way ANOVA with Sigmaplot 13. The complete analyses from all two-way repeated measures ANOVAs can be found in the electronic supplementary material, tables S1 and S2.

For data obtained by indirect calorimetry (including kcals consumed, EE, RER, CO and lipid oxidation), a two-sided mixed-model analysis was performed in R. For the mixed-model analyses (electronic supplementary material, tables S3–S6), we quantified the differences among the treatment groups by applying a linear mixed model to the full 15 d time courses for each of the following measurements: RER, LO, CO, locomotor activity, kcals consumed and kcals burned (oxidized). Each measurement was averaged using hourly bins for each mouse in each session. By using a mixed model, we were able to adjust for dependency of within-mouse observations. The model included random intercept and fixed effects for four treatment sessions: (i) day (treated as a factor variable and defined as 10.00 on 1 day to 9.59 on the next day); (ii) hour (treated as a factor variable); (iii) an interaction between treatment and day; and (iv) an interaction between treatment and hour. Pairwise mean differences were estimated from the integrated analysis of the mixed model.

## Data accessibility

The authors declare that all the data that support the findings of this study are available within the paper (and its electronic supplementary material information files). All data are available in the main text or the electronic supplementary material.

## References

[1] Hatori M et al. 2012 Time-restricted feeding without reducing caloric intake prevents metabolic diseases in mice fed a high-fat diet. Cell Metab. **15**, 848-860. (10.1016/j.cmet.2012.04.019) 22608008 PMC3491655

[2] Qian J, Morris CJ, Caputo R, Garaulet M, Scheer FAJL. 2018 Ghrelin is impacted by the endogenous circadian system and by circadian misalignment in humans. Int. J. Obesity **43**, 1644-1649. (10.1038/s41366-018-0208-9) PMC6424662 30232416

[3] Scheer FAJL, Hilton MF, Mantzoros CS, Shea SA. 2009 Adverse metabolic and cardiovascular consequences of circadian misalignment. Proc. Natl Acad. Sci. USA **106**, 4453-4458. (10.1073/pnas.0808180106) 19255424 PMC2657421

[4] Rudic RD, McNamara P, Curtis A-M, Boston RC, Panda S, Hogenesch JB, Fitzgerald GA. 2004 BMAL1 and CLOCK, two essential components of the circadian clock, are involved in glucose homeostasis. PLoS Biol. **2**, e377. (10.1371/journal.pbio.0020377) 15523558 PMC524471

[5] Panda S. 2018 The circadian code, especially pp. 98–99 and p. 200. New York, NY: Rodale Books.

[6] Manoogian ENC, Chaix A, Panda S. 2019 When to eat: the importance of eating patterns in health and disease. J. Biol. Rhythms **34**, 579-581. (10.1177/0748730419892105) 31813351 PMC7213043

[7] McHill AW et al. 2017 Later circadian timing of food intake is associated with increased body fat. Am. J. Clin. Nutr. **106**, 1213-1219. (10.3945/ajcn.117.161588) 28877894 PMC5657289

[8] Kelly KP et al. 2020 Eating breakfast and avoiding late-evening snacking sustains lipid oxidation. PLoS Biol. **18**, e3000622. (10.1371/journal.pbio.3000622) 32108181 PMC7046182

[9] Sato M et al. 2011 Acute effect of late evening meal on diurnal variation of blood glucose and energy metabolism. Obesity Res. Clin. Practice **5**, e220-e228. (10.1016/j.orcp.2011.02.001) 24331104

[10] Hibi M et al. 2013 Nighttime snacking reduces whole body fat oxidation and increases LDL cholesterol in healthy young women. AJP: Regul. Integr. Comp. Physiol. **304**, R94-R101. (10.1152/ajpregu.00115.2012) 23174861

[11] Nas A, Mirza N, Hägele F, Kahlhöfer J, Keller J, Rising R, Kufer TA, Bosy-Westphal A. 2017 Impact of breakfast skipping compared with dinner skipping on regulation of energy balance and metabolic risk. Am. J. Clin. Nutr. **105**, 1351-1361. (10.3945/ajcn.116.151332) 28490511

[12] Pan A, Schernhammer ES, Sun Q, Hu FB. 2011 Rotating night shift work and risk of type 2 diabetes: two prospective cohort studies in women. PLoS Med. **8**, e1001141. (10.1371/journal.pmed.1001141) 22162955 PMC3232220

[13] Arble DM, Bass J, Laposky AD, Vitaterna MH, Turek FW. 2009 Circadian timing of food intake contributes to weight gain. Obesity **17**, 2100-2102. (10.1038/oby.2009.264) 19730426 PMC3499064

[14] Bray MS, Tsai J-Y, Villegas-Montoya C, Boland BB, Blasier Z, Egbejimi O, Kueht M, Young ME. 2010 Time-of-day-dependent dietary fat consumption influences multiple cardiometabolic syndrome parameters in mice. Int. J. Obesity **34**, 1589-1598. (10.1038/ijo.2010.63) PMC3021134 20351731

[15] Haraguchi A, Aoki N, Ohtsu T, Ikeda Y, Tahara Y, Shibata S. 2014 Controlling access time to a high-fat diet during the inactive period protects against obesity in mice. Chronobiol. Int. **31**, 935-944. (10.3109/07420528.2014.931413) 24984029

[16] Oosterman JE, Foppen E, van der Spek R, Fliers E, Kalsbeek A, la Fleur SE. 2015 Timing of fat and liquid sugar intake alters substrate oxidation and food efficiency in male Wistar rats. Chronobiol. Int. **32**, 289-298. (10.3109/07420528.2014.971177) 25317718

[17] Chaix A, Zarrinpar A, Miu P, Panda S. 2014 Time-restricted feeding is a preventative and therapeutic intervention against diverse nutritional challenges. Cell Metab. **20**, 991-1005. (10.1016/j.cmet.2014.11.001) 25470547 PMC4255155

[18] Adamovich Y et al. 2014 Circadian clocks and feeding time regulate the oscillations and levels of hepatic triglycerides. Cell Metab. **19**, 319-330. (10.1016/j.cmet.2013.12.016) 24506873 PMC4261230

[19] Kobayashi F, Ogata H, Omi N, Nagasaka S, Yamaguchi S, Hibi M, Tokuyama K. 2014 Effect of breakfast skipping on diurnal variation of energy metabolism and blood glucose. Obesity Res. Clin. Practice **8**, e249-e257. (10.1016/j.orcp.2013.01.001) 24847666

[20] Ravussin E, Beyl RA, Poggiogalle E, Hsia DS, Peterson CM. 2019 Early time-restricted feeding reduces appetite and increases fat oxidation but does not affect energy expenditure in humans. Obesity **27**, 1244-1254. (10.1002/oby.22518) 31339000 PMC6658129

[21] Kuroda H et al. 2012 Meal frequency patterns determine the phase of mouse peripheral circadian clocks. Sci. Rep. **2**, 711. (10.1038/srep00711) 23050095 PMC3464454

[22] Luque RM, Park S, Kineman RD. 2007 Severity of the catabolic condition differentially modulates hypothalamic expression of growth hormone-releasing hormone in the fasted mouse: potential role of neuropeptide Y and corticotropin-releasing hormone. Endocrinology **148**, 300-309. (10.1210/en.2006-0592) 17038558

[23] Namvar S, Gyte A, Denn M, Leighton B, Piggins HD. 2016 Dietary fat and corticosterone levels are contributing factors to meal anticipation. Amer. J. Physiol. Regul. Integr. Comp. Physiol. **310**, R711-R723. (10.1152/ajpregu.00308.2015) 26818054 PMC4867411

[24] Yukimura Y, Bray GA. 1978 Effects of adrenalectomy on body weight and the size and number of fat cells in the Zucker (fatty) rat. Endocr. Res. Commun. **5**, 189-198. (10.1080/07435807809083752) 747998

[25] Wittmers LE, Haller EW. 1983 Effect of adrenalectomy on the metabolism of glucose in obese (C57 B1/6 J ob/ob) mice. Metabolism **32**, 1093-1100. (10.1016/0026-0495(83)90054-9) 6645960

[26] Gill S, Panda S. 2015 A smartphone app reveals erratic diurnal eating patterns in humans that can be modulated for health benefits. Cell Metab. **22**, 789-798. (10.1016/j.cmet.2015.09.005) 26411343 PMC4635036

[27] Kohsaka A, Laposky AD, Ramsey KM, Estrada C, Joshu C, Kobayashi Y, Turek FW, Bass J. 2007 High-fat diet disrupts behavioral and molecular circadian rhythms in mice. Cell Metab. **6**, 414-421. (10.1016/j.cmet.2007.09.006) 17983587

[28] Pendergast JS, Branecky KL, Yang W, Ellacott KLJ, Niswender KD, Yamazaki S. 2013 High-fat diet acutely affects circadian organisation and eating behavior. Eur. J. Neurosci. **37**, 1350-1356. (10.1111/ejn.12133) 23331763 PMC3645495

[29] Rose AJ, Vegiopoulos A, Herzig S. 2010 Role of glucocorticoids and the glucocorticoid receptor in metabolism: insights from genetic manipulations. J. Steroid Biochem. Mol. Biol. **122**, 10-20. (10.1016/j.jsbmb.2010.02.010) 20170729

[30] Le Minh N, Damiola F, Tronche F, Schütz G, Schibler U. 2001 Glucocorticoid hormones inhibit food-induced phase-shifting of peripheral circadian oscillators. EMBO J. **20**, 7128-7136. (10.1093/emboj/20.24.7128) 11742989 PMC125339

[31] Coomans CP et al. 2013 Detrimental effects of constant light exposure and high-fat diet on circadian energy metabolism and insulin sensitivity. FASEB J. **27**, 1721-1732. (10.1096/fj.12-210898) 23303208

[32] Shimizu H, Hanzawa F, Kim D, Sun S, Laurent T, Umeki M, Ikeda S, Mochizuki S, Oda H. 2018 Delayed first active-phase meal, a breakfast-skipping model, led to increased body weight and shifted the circadian oscillation of the hepatic clock and lipid metabolism-related genes in rats fed a high-fat diet. PLoS ONE **13**, e0206669. (10.1371/journal.pone.0206669) 30379940 PMC6209334

[33] Wilkinson CW, Shinsako J, Dallman MF. 1979 Daily rhythms in adrenal responsiveness to adrenocorticotropin are determined primarily by the time of feeding in the rat. Endocrinology **104**, 350-359. (10.1210/endo-104-2-350) 221174

[34] Frayn KN. 1983 Calculation of substrate oxidation rates in vivo from gaseous exchange. J. Appl. Physiol.: Respir. Environ. Exercise Physiol. **55**, 628-634. (10.1152/jappl.1983.55.2.628) 6618956

[35] Hall KD et al. 2016 Energy expenditure and body composition changes after an isocaloric ketogenic diet in overweight and obese men. Amer. J. Clin. Nutrition **104**, 324-333. (10.1111/obr.12399) 27385608 PMC4962163

[36] Baron KG, Reid KJ, Kern AS, Zee PC. 2011 Role of sleep timing in caloric intake and BMI. Obesity (Silver Spring) **19**, 1374-1381. (10.1038/oby.2011.100) 21527892

[37] Jakubowicz D, Froy O, Wainstein J, Boaz M. 2012 Meal timing and composition influence ghrelin levels, appetite scores and weight loss maintenance in overweight and obese adults. Steroids **77**, 323-331. (10.1016/j.steroids.2011.12.006) 22178258

[38] Garaulet M, Gómez-Abellán P, Alburquerque-Béjar JJ, Lee YC, Ordovás JM, Scheer FA. 2013 Timing of food intake predicts weight loss effectiveness. Int. J. Obes. (Lond) **37**, 604-611. (10.1038/ijo.2012.229) 23357955 PMC3756673

[39] Jakubowicz D, Barnea M, Wainstein J, Froy O. 2013 High caloric intake at breakfast vs. dinner differentially influences weight loss of overweight and obese women. Obesity (Silver Spring) **21**, 2504-2512. (10.1002/oby.20460) 23512957

